# Selenium for preventing cancer

**DOI:** 10.1590/S1516-31802012000100013

**Published:** 2012-02-13

**Authors:** Gabriele Dennert, Marcel Zwahlen, Maree Brinkman, Marco Vinceti, Maurice P. A. Zeegers, Markus Horneber

## Abstract

**BACKGROUND:**

Selenium is a trace element essential to humans. Higher selenium exposure and selenium supplements have been suggested to protect against several types of cancers.

**OBJECTIVE:**

Two research questions were addressed in this review: What is the evidence for: 1. an aetiological relationship between selenium exposure and cancer risk in women and men?; 2. the efficacy of selenium supplementation for cancer prevention in women and men?

**SEARCH STRATEGY:**

We searched electronic databases and bibliographies of reviews and included publications.

**SELECTION CRITERIA:**

We included prospective observational studies to answer research question (a) and randomised controlled trials (RCTs) to answer research question (b).

**DATA COLLECTION AND ANALYSIS:**

We conducted random effects meta-analyses of epidemiological data when five or more studies were retrieved for a specific outcome. We made a narrative summary of data from RCTs.

**MAIN RESULTS:**

We included 49 prospective observational studies and six RCTs. In epidemiologic data, we found a reduced cancer incidence (summary odds ratio, OR, 0.69; 95% confidence interval, CI, 0.53 to 0.91) and mortality (OR 0.55, 95% CI 0.36 to 0.83) with higher selenium exposure. Cancer risk was more pronouncedly reduced in men (incidence: OR 0.66, 95% CI 0.42 to 1.05) than in women (incidence: OR 0.90, 95% CI 0.45 to 1.77). These findings have potential limitations due to study design, quality and heterogeneity of the data, which complicated the interpretation of the summary statistics.

The RCTs found no protective efficacy of selenium yeast supplementation against non-melanoma skin cancer or L-selenomethionine supplementation against prostate cancer. Study results for the prevention of liver cancer with selenium supplements were inconsistent and studies had an unclear risk of bias. The results of the Nutritional Prevention of Cancer Trial (NPCT) and SELECT raised concerns about possible harmful effects of selenium supplements.

**AUTHORS’ CONCLUSIONS:**

No reliable conclusions can be drawn regarding a causal relationship between low selenium exposure and an increased risk of cancer. Despite evidence for an inverse association between selenium exposure and the risk of some types of cancer, these results should be interpreted with care due to the potential limiting factors of heterogeneity and influences of unknown biases, confounding and effect modification.

The effect of selenium supplementation from RCTs yielded inconsistent results. To date, there is no convincing evidence that selenium supplements can prevent cancer in men, women or children.

This is the abstract of a Cochrane Review published in the Cochrane Database of Systematic Reviews (CDSR) 2011, Issue 5, Art n^º^ CD005195. DOI:10.1002/14651858.CD005195.pub2 (http://www.thecochranelibrary.com). For full citation and authors details, see reference 1. 

For Latin America and the Caribbean, the full text is freely available from: http://www.cochranejournalclub.com/selenium-preventing-cancer-clinical/pdf/CD005195.pdf
